# Prevalence and correlates of childhood anemia in the MINA-Brazil birth cohort study

**DOI:** 10.11606/s1518-8787.2023057005637

**Published:** 2024-02-01

**Authors:** Marly A Cardoso, Bárbara H. Lourenço, Alicia Matijasevich, Marcia C Castro, Marcelo U Ferreira

**Affiliations:** I Universidade de São Paulo Faculdade de Saúde Pública Departamento de Nutrição São Paulo SP Brasil Universidade de São Paulo. Faculdade de Saúde Pública. Departamento de Nutrição. São Paulo, SP, Brasil; II Universidade de São Paulo Faculdade de Medicina Departamento de Medicina Preventiva São Paulo SP Brasil Universidade de São Paulo. Faculdade de Medicina. Departamento de Medicina Preventiva. São Paulo, SP, Brasil; III Harvard T.H. Chan School of Public Health Department of Global Health and Population Boston MA United States of America Harvard T.H. Chan School of Public Health. Department of Global Health and Population. Boston, MA, United States of America; IV Universidade de São Paulo Instituto de Ciências Biomédicas Departamento de Parasitologia São Paulo SP Brasil Universidade de São Paulo, Instituto de Ciências Biomédicas. Departamento de Parasitologia. São Paulo, SP, Brasil

**Keywords:** Anemia, Iron Deficiency, Malaria; Risk Factors, Cohort Studies

## Abstract

**OBJECTIVE:**

This study aimed to describe the prevalence and predictors of childhood anemia in an Amazonian population-based birth cohort study.

**METHODS:**

Prevalence of maternal anemia was estimated at delivery (hemoglobin [Hb] concentration < 110 g/L) in women participating in the MINA-Brazil birth cohort study and in their children, examined at ages one, two (Hb < 110 g/L), and five (Hb < 115 g/L). Moreover, ferritin, soluble transferrin receptor, and C-reactive protein concentrations were measured in mothers at delivery and in their 1- and 2-year-old children to estimate the prevalence of iron deficiency and its contribution to anemia, while adjusting for potential confounders by multiple Poisson regression analysis (adjusted relative risk [RR_a_]).

**RESULTS:**

The prevalence 95% confidence interval (CI) of maternal anemia, iron deficiency, and iron-deficiency anemia at delivery were 17.3% (14.0–21.0%), 42.6% (38.0–47.2%), and 8.7% (6.3–11.6)%, respectively (n = 462). At one year of age (n = 646), 42.2% (38.7–45.8%) of the study children were anemic, 38.4% (34.6–42.3%) were iron-deficient, and 26.3 (23.0–29.9) had iron-deficiency anemia. At two years of age (n = 761), these values decreased to 12.8% (10.6–15.2%), 18.1% (15.5–21.1%), and 4.1% (2.8–5.7%), respectively; at five years of age (n = 655), 5.2% (3.6–7.2%) were anemic. Iron deficiency (RR_a_ = 2.19; 95%CI: 1.84–2.60) and consumption of ultra-processed foods (UPF) (RR_a_ = 1.56; 95%CI: 1.14–2.13) were significant contributors to anemia at 1 year, after adjusting for maternal schooling. At 2 years, anemia was significantly associated with maternal anemia at delivery (RR_a_: 1.67; 95%CI: 1.17–2.39), malaria since birth (2.25; 1.30–3.87), and iron deficiency (2.15; 1.47–3.15), after adjusting for children's age and household wealth index.

**CONCLUSIONS:**

Anemia continues to be highly prevalent during pregnancy and early childhood in the Amazon. Public health policies should address iron deficiency, UPF intake, maternal anemia, and malaria to prevent and treat anemia in Amazonian children.

## INTRODUCTION

Nearly one-third of the world's population is estimated to be anemic, with increased morbidity and mortality, decreased work productivity, and impaired child neurodevelopment^1^. Infants, young children, and pregnant women are among the populations most vulnerable to anemia worldwide^2^. From 2000 to 2019, the prevalence of anemia in children aged 6–59 months decreased from 48% to 40% worldwide and from 30% to 20% in Latin America and the Caribbean; however, the burden of childhood anemia remains staggering in many low- and middle-income countries^3^. Recent meta-analyses reveal regional disparities in the prevalence of childhood anemia in Brazil that are hidden behind pooled countrywide estimates^4,5^. For example, the Brazilian National Survey on Child Nutrition (ENANI-2019) estimated that 10% of the preschool children living in Brazilian metropolitan areas were anemic; however, in the North—the region that comprises most of the Amazon Basin of Brazil—, the prevalence reached 17%^6^. The corresponding estimates for children aged 6–23 months were 20% countrywide and 30% in the North^6^.

Iron deficiency accounts for nearly 50% of the global burden of childhood anemia^7,8^. For the primary prevention of iron-deficiency anemia in young children, the World Health Organization (WHO) recommends exclusive breastfeeding (EBF) for six months and continued breastfeeding (BF) until 2 years or older, along with adequate complementary feeding with iron or multiple micronutrient supplements^9^. However, supplementation programs to prevent anemia have a relatively low coverage in most low- and middle-income countries^7^ and fail to address non-nutritional causes of childhood anemia, such as soil-transmitted helminthic infections, schistosomiasis, and malaria^2^.

This study focused on childhood anemia in the North of Brazil, the region with the highest prevalence of anemia in preschool children estimated in the most recent countrywide survey^6^. We described the prevalence of anemia during the first five years of follow-up of children participating in an ongoing population-based birth cohort study in the Amazon and identified independent predictors of anemia risk that may constitute potential targets for public health interventions.

## METHODS

### Study Design, Population, and Data Collection

The Maternal and Child Health and Nutrition in Acre, Brazil (MINA-Brazil) birth cohort study started in 2015 with 1,246 mother-child pairs in the Western Amazonian city of Cruzeiro do Sul (CZS), Acre State. It aimed to characterize the effect of a wide range of early exposures on child health^10^. The infant mortality rate in CZS was estimated at 10.8 deaths per 1,000 live births in 2015 and 10.6 per 1,000 in 2020^11^. CZS experiences year-round malaria transmission, with nearly 90% of all malarial infections in young children being due to *Plasmodium vivax*^12^. The annual malaria incidence (API; number of laboratory-confirmed cases per 1,000 people per year)—one of the highest among Brazilian municipalities—was estimated at 231.9 in CZS in 2016^12^. Mother-baby pairs were enrolled at pregnancy in public antenatal clinics, or at birth in the Women and Children's Hospital of Juruá Valley, the only maternity hospital of CZS. Informed consent forms were obtained at enrollment from mothers or caregivers (in the case of teenage mothers). The research protocols were approved by the Research Ethics Committee of the Faculdade de Saúde Pública da Universidade de São Paulo, Brazil (# 872.613, 2014; # 2.358.129, 2017).

During pregnancy, clinical and laboratory assessments were conducted by the research team in a subsample (n = 557) of the entire cohort (n =1,246). At delivery, the following data from both interviews and medical records were obtained: sociodemographic (maternal schooling, mothers’ self-reported skin color, whether the family was supported by the Bolsa Família Program, a conditional cash transfer program, and an assets-based wealth index used as a proxy of socioeconomic status) and maternal and perinatal data (such as parity, number of antenatal care visits, smoking and alcohol consumption during pregnancy, maternal hemoglobin concentration and body weight at delivery, gestational age and type of delivery, child's sex, and birthweight). From the prenatal card, information on maternal height (m) and pre-pregnancy weight (kg) were collected for all participants. Pre-pregnancy body mass index (BMI) was categorized as underweight (< 18.5 kg/m^2^), normal weight (18.5–24.9 kg/m^2^), overweight (25.0–29.9 kg/m^2^), or obese (≥ 30.0 kg/m^2^) as defined by the WHO. The difference between weight at delivery and pre-pregnancy weight was used to estimate maternal gestational weight gain (GWG). Based on pre-pregnancy BMI categories, GWG was classified as insufficient, adequate, or excessive following the Institute of Medicine 2009 guidelines^13^.

After birth, clinical assessments with blood sample collection were performed at healthcare units when children were aged one, two, and five years, as described elsewhere^10^. Anthropometric measurements were performed in duplicate using standardized procedures. Child anthropometric indexes in z-scores were calculated according to age and sex following the WHO Child Growth Standards; stunting and overweight were defined as length or height for age in z-score < −2 and body mass index (BMI)-to-age > 2 z-scores, respectively^14^.

At each childhood follow-up visit, structured questionnaires were administered to mothers or guardians to update data, including infant feeding practices, morbidities since birth, and other characteristics. Mothers reported whether the child was being breastfed (yes or no) and, if not, the age of weaning. Children who received breast milk with no other food or drink, except prescribed medicines, oral rehydration solutions, vitamins, and minerals, were considered exclusively breastfed. Continued breastfeeding (BF) was estimated in days and then classified as prolonged BF when ≥ 365 days. At the 1- and 2-year follow-up visits, information on complementary feeding was assessed by a structured food frequency questionnaire on the intake of foods and drinks in the previous day, as detailed elsewhere^15^. The following ultra-processed foods (UPF) were included in the questionnaire: industrialized yogurt, artificial fruit juice, soft drinks, candies, cookies, packaged savory snacks, hotdogs, and instant noodles, and “other UPF” (chocolate drinks, ice cream, jelly, cake, and industrialized soup). Then, the prevalence of UPF consumption was estimated based on the intake of at least one food from the category during the previous day.

Venous blood samples (10 mL for third-trimester pregnant women and 5 mL for children) were collected for measuring biochemical nutritional indicators during pregnancy and at the 1- and 2-year follow-up assessments; at the 5-year follow-up, only blood hemoglobin (Hb) concentration was measured. A Hb concentration < 110 g/L, measured in antenatal clinics using a portable Hemocue (Hb301; Angelholm, Sweden) hemoglobinometer defined anemia in pregnancy^16^. A Labtest SDH-20 cell counter (Labtest, Lagoa Santa, Brazil) was used to measure mothers’ Hb levels at the maternity ward. . An ABX Micro 60 cell counter (Horiba, Montpellier, France) was used to measure children's Hb concentrations at the 1- and 2-year follow-up visits; whereas at the 5-year follow-up, a portable Hemocue hemoglobinometer was used. Blood samples were protected from light and centrifuged within two hours after collection; serum and plasma samples were frozen at −20°C, shipped on dry ice to São Paulo, and kept at −70°C until further analyses. Plasma ferritin and soluble transferrin receptor concentrations were measured using enzyme immunoassay (Ramco, Houston, TX, USA). Liquid chromatography (HPLC) was used to measure serum concentrations of retinol as described elsewhere^17^. Fluoroimmunoassay (PerkinElmer; Wallac Oy, Turku, Finland) was used to measure serum folate concentrations. An IMMAGE Immunochemistry System (Beckman Coulter, Brea, CA, USA) was used to determine C-reactive protein. Analyses were subject to internal and external quality control with routine use of blind samples for each run, with coefficients of variation < 7%.

Cut-off values established by the WHO were adopted to define vitamin deficiencies: serum retinol concentrations < 0.7 μmol/L for vitamin A deficiency (VAD) and < 1.05 μmol/L for vitamin A insufficiency (VAI)^18^, and serum folate concentration < 3 ng/mL (pregnant women) or < 4 ng/mL (children) for folate deficiency^19^. Iron deficiency (ID) was defined as plasma ferritin concentration < 12 μg/L (children) or < 15 μg/L (pregnant women), combined with a soluble transferrin receptor concentration > 8.3 mg/L, or plasma ferritin < 30 μg/L combined with a C-reactive protein concentration ≥ 5 mg/L (suggestive of acute inflammation)^20^.

Malaria during pregnancy was diagnosed by microscopy during visits to health units and/or by real-time polymerase chain reaction (PCR) at delivery^21^. Malaria in young children was diagnosed by microscopy during visits to health units. Case notifications during pregnancy and the first two years of life were retrieved from the electronic database of the Brazilian Ministry of Health (SIVEP Malaria) using a previously described linkage strategy^12^.

Anemia, nutritional deficiencies, and malaria diagnosed during the follow-up visits were treated by research physicians. Children with anemia were prescribed ferrous sulfate (dose: 3–6 mg of elemental iron/kg per day up to 60 mg/day for four months) for presumptive treatment of iron deficiency, but adherence to this treatment was not evaluated.

### Main Outcome Measures

Childhood anemia was defined as Hb < 110 g/L for children < 5 years. At the 5-year follow-up visit, Hb < 115 g/L defined anemia for children ≥ 5 years. Severe anemia was classified as Hb < 70 g/L and < 80 g/L for children < 5 and ≥ 5 years, respectively^16^.

### Data Analysis

Maternal and child characteristics were described using absolute frequencies and proportions (%) with 95% confidence intervals (CI). Mean and SD for age and median values and interquartile ranges (IQR, 25^th^ and 75^th^ percentiles) for biochemical nutritional indicators were calculated. Pearson χ^2^ or Fisher exact tests were used to compare proportions.

**Figure 1:**
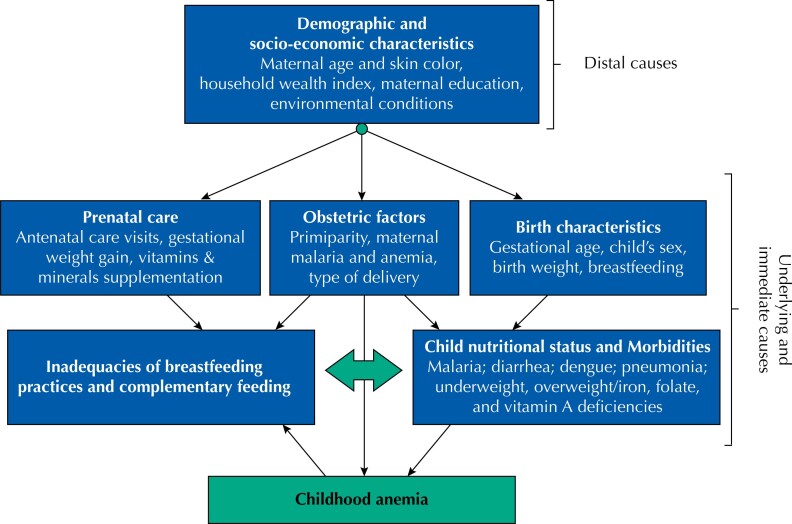
Causal hierarchical approach for childhood anemia based on the available variables in the MINA-Brazil birth cohort study, adapted from previous conceptual frameworks^7,22^

Relative risks (RR) with 95%CI were estimated using multiple Poisson regression models with robust variance to identify factors associated with childhood anemia at each follow-up visit and with “persistent anemia” (children who were anemic in both the 1-year and the 2-year visits). Multiple adjusted linear regression coefficients (β) were also calculated to describe predictors for concentrations of Hb (dependent variable) at each follow-up assessment. Variables associated with the outcomes at a significance level < 20% in unadjusted analysis were entered in multiple regression models. A hierarchical approach was used based on conceptual frameworks^7,22^ to select distal (demographic and socio-economic factors), underlying (antenatal care, obstetric and birth characteristics), and immediate (child feeding practices, nutritional status, and morbidities) determinants of childhood anemia in the final adjusted models (Figure 1). At each level of determination, covariates were retained in the model if they were associated with the outcome at P < 0.10 and/or for ordinal variables with more than two categories if they followed a dose-response pattern. Missing values in categorical covariates were maintained in the model by creating a new missing-value category. All P-values reported are two-tailed and the significant level was set at P < 0.05. Statistical analyses were performed using Stata version 15.0 (StataCorp, College Station, TX, USA).

## RESULTS

**Figure 2:**
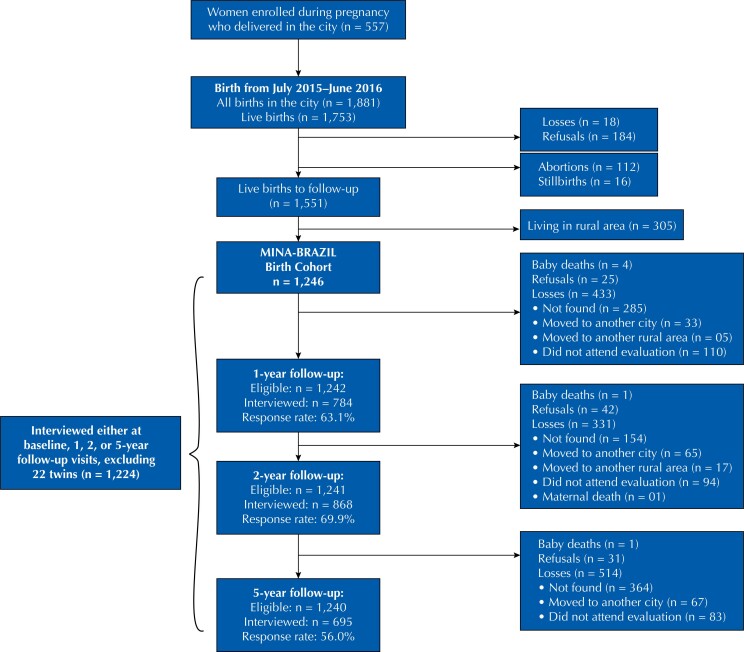
Flowchart of the MINA-Brazil birth cohort study since pregnancy to 5-year follow-up visits.

At baseline, we obtained data from 1,246 participants of the MINA Brazil cohort. After exclusion of 22 twins, a total of 1,224 mother-child pairs were eligible for the present analysis. Of these, 79 missed all post-natal follow-up visits and six children died up to five years of age (Figure 2). Children participating from birth to different follow-up assessments and those lost to follow-up over time until the 5-year assessment (n = 514) had similar perinatal characteristics regarding sex, gestational age, preterm birth, and birth weight, but differed significantly in the proportion of children from poorest families (36.2% *versus* 44.1%) and of mothers with ≤ 9 years of schooling (26.4% *versus* 47.5%), respectively, with P < 0.01 (c^2^ test) for both.

The overall prevalence of childhood anemia declined from 42% to 13% and 5% at 1-, 2-, and 5-year assessments, respectively. A total of two cases (0.3%) of severe anemia (Hb < 70 g/L) were observed at the 1-year follow-up; the prevalence of moderate anemia was 14.2%, 1.5%, and 1.5% at 1, 2, and 5 years of age, respectively (Table 1). In crude analysis, the lowest wealth index, lowest maternal age, lowest schooling, prolonged breastfeeding, and consumption of UPF were significantly associated with anemia at one year of age. At two years, children in the lowest wealth index, born to a multiparous mother, with < 6 antenatal care visits, and history of one or more malaria infections since birth were at higher risk for anemia. At five years, only prolonged breastfeeding was significantly associated with lower risk of anemia.

**Table 1:** Characteristics of the participants in the MINA-Brazil birth cohort study regarding childhood anemia at follow-up visits.

Characteristic			Child follow-up visits						
			Totals^a^ (%)	Anemia^b^ at 1 y (n = 768)		Anemia^b^ at 2 y (n = 846)		Anemia at 5 y (n = 655)	
				n (%)	p-value	n (%)	p-value	n (%)	p-value
Overall			1,224	324 (42.2)	-	108 (12.8)	-	31 (5.2)	-
Baseline characteristics									
	Maternal age at delivery (years)				0.009		0.893		0.832
		< 19	229 (18.7)	65 (52.9)		16 (12.4)		6 (5.6)	
		≥ 19	995 (81.3)	259 (40.2)		92 (12.8)		28 (5.1)	
	Maternal self-reported skin color				0.913		0.559		0.233
		White	149 (12.5)	39 (41.9)		12 (10.9)		2 (2.5)	
		Black, Mixed-race, Indigenous people, and Yellow	1,042 (87.5)	282 (42.5)		93 (12.9)		32 (5.7)	
	Wealth index in tertiles				< 0.001		0.004		0.768
		Lowest	400 (33.6)	113 (52.1)		43 (18.8)		9 (5.8)	
		Second	392 (32.9)	121 (46.5)		31 (10.8)		10 (4.4)	
		Highest	399 (33.5)	87 (31.2)		31 (9.8)		15 (5.8)	
	Maternal schooling (years)				< 0.001		0.181		0.647
		≤ 9	422 (35.5)	123 (54.7)		36 (15.1)		10 (6.0)	
		> 9	768 (64.5)	197 (37.2)		69 (11.7)		24 (5.1)	
	Bolsa Familia program				0.032		0.064		0.233
		No	720 (60.5)	188 (39.5)		57 (11.0)		23 (5.6)	
		Yes	471 (39.6)	133 (47.5)		48 (15.4)		11 (4.7)	
	Antenatal care visits				0.185		0.047		0.455
		< 6	288 (23.7)	66 (47.1)		29 (17.5)		4 (3.7)	
		≥ 6	926 (76.3)	256 (41.0)		79 (11.7)		30 (5.5)	
	Malaria during pregnancy				0.358		0.909		0.354
		No	1,142 (93.3)	298 (41.7)		101 (12.8)		31 (5.0)	
		Yes	82 (6.7)	26 (48.2)		7 (12.3)		3 (8.6)	
	Gestational weight gain^c^				0.434		0.760		0.688
		Insufficient	341 (31.3)	93 (44.1)		27 (11.3)		7 (3.9)	
		Adequate	392 (36.0)	101 (38.7)		35 (12.4)		12 (5.5)	
		Excessive	357 (32.8)	110 (43.1)		37 (13.4)		12 (5.6)	
	Parity				0.159		0.034		0.200
		Primiparous	498 (41.8)	126 (39.5)		33 (9.7)		16 (4.3)	
		Multiparous	693 (58.2)	195 (44.6)		72 (44.7)		18 (6.6)	
	Type of delivery				0.971		0.432		0.570
		Vaginal	660 (53.9)	169 (42.3)		61 (13.6)		19 (5.7)	
		Cesarean	564 (46.1)	155 (42.1)		47 (11.8)		15 (4.7)	
	Preterm birth (gestational age) (weeks)				0.148		0.310		0.670
		No (≥ 37)	1,120 (91.5)	304 (42.9)		102 (13.1)		32 (5.3)	
		Yes (< 37)	104 (8.5)	20 (33.3)		6 (8.8)		2 (3.9)	
	Birth weight (grams)				0.486		0.485		0.706
		2,500 - < 4,000	1,061 (86.8)	288 (42.9)		94 (12.9)		28 (4.9)	
		< 2,500	86 (7.0)	18 (36.7)		5 (8.6)		3 (7.3)	
		≥ 4,000	76 (6.2)	17 (36.2)		9 (16.1)		3 (6.8)	
Child characteristics during follow-up									
	Exclusive breastfeeding ≥ 90 days				0.760		0.295		0.080
		No	691 (67.0)	198 (41.7)		68 (13.7)		25 (6.5)	
		Yes	340 (33.0)	125 (42.8)		30 (11.1)		7 (3.2)	
	Prolonged exclusive breastfeeding (≥ 365 days)				< 0.001		0.394		0.039
		No	318 (34.9)	63 (28.3)		29 (11.3)		15 (7.8)	
		Yes	593 (65.1)	216 (46.2)		79 (13.4)		16 (3.9)	
	Malaria in the first 2 years				0.338		0.016		0.386
		No	808 (94.6)	313 (41.9)		97 (12.1)		28 (5.0)	
		Yes	46 (5.4)	11 (52.4)		11 (24.4)		2 (9.1)	
	Consumption of beans^d^				0.362		0.056		0.468
		No	-	147 (42.6)		69 (14.9)		14 (4.4)	
		Yes	-	118 (39.1)		39 (10.4)		15 (5.7)	
	Consumption of meats^d^				0.686		0.170		0.473
		No	-	77 (41.0)		17 (17.2)		2 (3.2)	
		Yes	-	246 (42.6)		91 (12.3)		28 (5.4)	
	Consumption of ultra-processed foods^d^,^e^				0.007		0.746		0.531
		No	-	29 (29.6)		7 (11.5)		1 (2.9)	
		Yes	-	295 (44.0)		101 (12.9)		29 (5.3)	

a Totals differ due to missing values; P-values for Pearson χ^2^ test or Fisher exact test (cell count < 5).

b World Health Organization classification criteria for anemia (2011): blood hemoglobin concentrations < 110 g/L for children under 5 years and < 115g/L for children ≥ 5 years.

c Institute of Medicine Guidelines, 2009.

d Food frequency intake in the previous day.

e Consumption of at least one ultra-processed food.

Table 2 shows the mean values of age (SD), frequencies of the use of iron and/or vitamin supplements, prevalence (95%CI) of nutritional deficiencies, and interquartile ranges (IQR) of biochemical parameters at different waves of the entire cohort. Among pregnant and parturient women, the most frequently used supplements were iron (30–60 mg/day) and folic acid (5 mg/day), following national antenatal care guidelines. At delivery, similar frequencies (around 30%) of underweight and excessive GWG were observed. Anemia was common among pregnant women (17%) and parturients (38%). Inadequacies of iron or vitamin A status were frequent among pregnant women and children under two years; folic acid deficiency was uncommon, with prevalence < 1% in mothers and children. Combined iron and vitamin A deficiencies were more common at two years (6.7%).

**Table 2:** Nutritional characteristics of the participants from the MINA-Brazil cohort study (n = 1,224).

Characteristic						Child follow-up visits					
		Pregnant women		Parturients		1 year		2 years		5 years	
		n = 557	Mean (SD)	n = 1,224	Mean (SD)	n = 774	Mean (SD) or %	n = 854	Mean (SD) or %	n = 682	Mean (SD) or %
Mean age (years or months)			24.8 y (6.5)		24.9 y (6.6)		12.7 m (0.7)		23.8 m (1.4)		63.4 m (2.7)
Female sex			-		-	404	52.2	427	50.0	336	49.3
Use of vitamin and/or mineral supplementation		n = 557	%	n = 1,224	%	n = 774	%	n = 854	%	n = 682	%
	Iron		69.1		65.9		9.4		3.5		0.4
	Multinutrients		38.1		42.6		32.7		26.4		27.1
	Folic acid (5 mg)		70.7		66.4		-		-		-
	Vitamin A		34.3		61.2		11.2		31.4		21.0
Prevalence of anemia and micronutrient deficiencies		n^a^	% (95%CI)	n	% (95%CI)	n	% (95%CI)	n	% (95%CI)	n	% (95%CI)
	Anemia^b^	469	17.3 (14.0–21.0)	1,156	37.7 (35.0–40.5)	768	42.2 (38.7–45.8)	846	12.8 (10.6–15.2)	655	5.2 (3.6–7.2)
	Iron deficiency^c^	463	42.6 (38.0–47.2)		-	646	38.4 (34.6–42.3)	761	18.1 (15.5–21.1)		-
	Iron deficiency anemia	462	8.7 (6.3–11.6)		-	646	26.3 (23.0–29.9)	761	4.1 (2.8–5.7)		-
	Vitamin A deficiency^d^	467	6.4 (4.4–9.0)		-	533	1.7 (0.8–3.2)	703	24.8 (21.6–28.1)		-
	Vitamin A insufficiency^d^	467	19.7 (16.2–23.6)		-	533	10.3 (7.9–13.2)	703	41.7 (38.0–45.4)		-
	Folic acid deficiency^e^	464	0.7 (0.1–1.9)		-	484	1.0 (0.3–2.4)	490	0.4 (0.1–1.50)		
	Combined iron and vitamin A deficiencies	461	2.8 (1.5–4.8)		-	532	0.8 (0.2–1.9)	699	6.7 (5.0–8.8)		-
	C-reactive protein ≥ 5 mg/L (acute inflammation cut-off)	465	0.35 (0.31–0.40)			641	12.9 (10.4– 15.8)	712	10.5 (8.4–13.0)		-
Biochemical parameter		n	Median (IQR)	n	Median (IQR)	n	Median (IQR)	n	Median (IQR)	n	Median (IQR)
	Hemoglobin (g/L)	469	119 (112–125)	1,156	113 (104–120)	768	112 (104–119)	846	121 (114–127)	655	128 (122–133)
	Plasma ferritin (μg/L)	463	17.0 (10.0–27.0)		-	639	21.0 (12.0–33.0)	725	31.0 (21.0–43.0)		-
	Serum retinol (μmol/L)	467	1.9 (1.2–2.7)		-	533	1.9 (1.4–2.8)	703	1.2 (0.7–1.7)		-
	Serum folic acid (ng/mL)	464	9.6 (7.1–13.4)		-	484	14.0 (11.0–18.2)	490	14.3 (11.3–18.8)		-

95%CI: 95% confidence interval; SD: standard deviation; IQR: interquartile ranges.

a Blood samples in the third gestational trimester (mean gestational age: 27.8, SD: 1.6, ranging from 24 to 34 weeks).

b WHO classification criteria for anemia (2011): hemoglobin concentrations < 110 g/L for pregnant women and children under 5 years, and < 115g/L for children ≥ 5 years.

c Iron deficiency: pregnant women, ferritin < 15 ug/L; children: ferritin < 12 ug/L and soluble transferrin receptor > 8.3 mg/L.

d Vitamin A deficiency < 0.7 μmol/L, vitamin A insufficiency < 1.05 μmol/L.

e Folic acid cut-off: pregnant women < 3.0 ng/mL; children < 4.0 ng/mL.

ID (adjusted RR [RR_a_] = 2.19; 95%CI: 1.84–2.60) and consumption of UPF (RR_a_ = 1.56; 95%CI: 1.14–2.13) were associated with elevated risk of anemia at one year, after adjustment for maternal schooling in multiple regression analysis (Table 3). At the 2-year follow-up visit, maternal anemia at delivery (RR_a_ = 1.67; 95%CI: 1.17–2.39), malaria in the first 2 years of life (RR_a_ = 2.25; 95%CI: 1.30–3.87), and ID (RR_a_ = 2.15; 95%CI: 1.47–3.15) were significantly associated with increased risk of anemia, after adjustment for child's age and household wealth index. Risk of persistent anemia in the 1- and 2-year follow-up visits (n = 53) was positively associated with male sex (RR_a_ = 1.87; 95%CI: 1.05–3.31), malaria during the first two years of life (RR_a_ = 4.30; 95%CI: 2.33–7.94), prolonged breastfeeding (RR_a_ = 2.48; 95%CI: 1.11–5.51), and vitamin A insufficiency (RR_a_ = 2.03; 95%CI: 1.16–3.54); consumption of meat emerged as a protective factor (RR_a_ = 0.52; 95%CI: 0.29– 0.95). At 5 years, only maternal anemia at delivery was positively associated with risk of anemia (RR_a_ = 2.32; 95%CI: 1.11– 4.84), whereas prolonged breastfeeding was inversely associated with risk of anemia (RR_a_ = 0.45; 95%CI: 0.22– 0.94).

**Table 3:** Multiple adjusted relative risk (RR_a_) for childhood anemia and adjusted regression coefficients (aβ) for predictors of hemoglobin values (Hb, g/L) in the MINA-Brazil cohort study.

Exposure		Anemia risk at the 1-y follow-up (anemics/total = 324/768)		Anemia risk at the 2-y follow-up (n = 846)				Anemia risk at the 5-y follow-up (anemics/total = 34/655)^a^	
				All children with anemia n = 108		Children with persistent anemia from 1 year to 2 years (n = 53)			
		RR_a_ (95%CI)	p-value	RR_a_ (95%CI)	p-value	RR_a_ (95%CI)	p-value	RR_a_ (95%CI)	p-value
Wealth index (tertiles)									
	Lowest	-	-	1		-	-	-	-
	Second	-	-	0.61 (0.39 to 0.95)	0.030	-	-	-	-
	Highest	-	-	0.96 (0.39 to 2.36)	0.029	-	-	-	-
Maternal schooling (years)									
	≤ 9	1		-	-	-	-	-	-
	10–12	0.83 (0.71 to 0.99)	0.034	-	-	-	-	-	-
	> 12	0.70 (0.53 to 0.91)	0.008	-	-	-	-	-	-
Child's age (months)		-	-	1.18 (1.06 to 1.32)	0.002	-	-	-	-
Male child		-	-	-	-	1.87 (1.05 to 3.31)	0.047	-	-
Maternal anemia at delivery		-	-	1.67 (1.17 to 2.39)	0.005	-	-	2.32 (1.11 to 4.84)	0.025
Prolonged breastfeeding (≥ 365 days)		-	-	-	-	2.48 (1.11 to 5.51)	0.001	0.45 (0.22 to 0.94)	0.032
UPF intake at 1 year		1.56 (1.14 to 2.13)	0.005	-	-	-	-	-	-
Dietary intake of meats		-	-	-	-	0.52 (0.29 to 0.95)	0.026		
Malaria in the first 2 years		-	-	2.25 (1.30 to 3.87)	0.003	4.38 (2.33 to 7.94)	<0.001	-	-
Iron deficiency		2.19 (1.84 to 2.60)	< 0.001	2.15 (1.47 to 3.15)	< 0.001	-	-	-	-
Vitamin A insufficiency		-	-	-	-	2.03 (1.16 to 3.54)	0.013	-	-
		**Hb predictors at 1-y** **follow-up**		**Hb predictors at 2-y follow-up**				**Hb predictors at 5-y** **follow-up**	
		**(R^2^ = 0.200)**		**(R^2^ = 0.095)**				**(R^2^ = 0.061)**	
		**aβ (95%CI)**	**p-value**	**aβ (95%CI)**	**p-value**			**aβ (95%CI)**	**p-value**
Wealth index (tertiles)									
	Lowest	-	-	Ref.			-		-
	Second	-	-	1.86 (0.13 to 3.60)	0.036		-	-	-
	Highest	-	-	2.68 (0.95 to 4.41)	0.002		-	-	-
Maternal schooling (years)									
	≤ 9	Ref.		-	-			-	-
	10–12	2.82 (0.74 to 4.91)	0.008	-	-		-	-	-
	> 12	3.84 (1.13 to 6.55)	0.006	-	-		-	-	-
Primiparous mother		-	-	1.92 (0.50 to 3.33)	0.008		-	-	-
Maternal anemia at delivery		-	-	−3.12 (−4.54 to −1.71)	< 0.001		-	−3.16 (−4.80 to −1.52)	< 0.001
Prolonged breastfeeding (≥ 365 days)		-	-	−	-		-	2.16 (0.39 to 3.93)	0.017
Malaria in the first 2 years		-	-	−4.15 (−7.25 to −1.05)	0.009		-	−9.26 (−13.56 to 4.97)	< 0.001
Consumption of UPF at 1 year		-	-	-	-		-	−2.47 (−4.94 to −0.01)	0.050
Consumption of beans group at 2 years		-	-	1.90 (0.51 to 3.30)	0.007		-	-	-
Iron deficiency		−9.55 (−11.44 to −7.67)	< 0.001	−4.18 (−5.96 to −2.40)	< 0.001		-	-	-
Vitamin A insufficiency		−3.26 (−6.25 to −0.27)	0.033	-	-		-	-	-

UPF: ultra-processed food.

a Of the 34 children with anemia, persistent anemic cases from 1 to 5 year were n = 8.

Predictors of Hb concentrations during childhood were also explored (Table 3). At one year, maternal schooling was positively associated with Hb concentration, whereas ID and VAI were negatively associated. At two years, children of families in the highest wealth stratum, born to primiparous mothers, and those who consumed beans had higher Hb concentrations, whereas maternal anemia at delivery, malaria in the first 2 years, and iron deficiency were negatively associated with Hb concentration. At five years, prolonged breastfeeding was positively associated with Hb concentration; conversely, maternal anemia, consumption of UPF at one year, and malaria in the first two years of life showed a negative association.

## DISCUSSION

In this study, we addressed some of the most likely causes of childhood anemia in the Amazon. We showed that iron deficiency (ID) is still very common, affecting 43% of local pregnant women and 38% of children at one year of age, but its contribution to anemia seems to vary with age. Accordingly, we diagnosed ID in 51% of pregnant women and 62% of the children aged one who were found to be anemic, which was observed in only 32% of the anemic children aged two. VAD and VAI were also common during pregnancy (6% and 20%, respectively) and in children aged two (25% and 42%, respectively), which was substantially less frequent in children aged one (2% and 10%, respectively), when childhood anemia is most prevalent. Evidence that VAI or VAD contributes to anemia in our population is twofold: we found a significant association between VAI and elevated risk of persistent anemia in early childhood, as well as a negative impact of VAI on Hb levels at the age of one year.

Children born to mothers who were anemic at delivery were more likely to be anemic at the ages of two and five after adjusting for potential confounders. Although this association is likely to be causal, residual confounding must also be considered since mother-child pairs sharing the same household are similarly exposed to poverty, food insecurity, and other key contributors to nutritional deficiencies and anemia. We highlight that recent evidence shows that anemia in pregnancy increases the risk of ID and anemia in infants, suggesting that the benefits of preventing anemia in pregnancy might extend to the offspring by reducing anemia risk in early childhood^23^. Our results reinforce the argument for routine iron supplementation in pregnancy as advocated by WHO in areas with a prevalence > 40%^16^.

Folate deficiency was rare among MINA-Brazil cohort participants, most likely due to folic acid fortification of wheat flour implemented in Brazil in 2004. Brazil has adopted the fortification of wheat and maize flours with 4.2 mg of iron and 150 mg of folic acid added per 100 g. However, the effectiveness of iron fortification of flour in preventing anemia seems to be poor among young children^24^. The transition to complementary foods as children begin to consume household diets is associated with insufficient iron intake, combined with low dietary intake of other micronutrients^7^. Thus, since 2011, the use of multiple micronutrients in powder (MNP) has been recommended by WHO as a home-based strategy to prevent and control childhood anemia. In 2014, the Brazilian Ministry of Health launched the Multiple Micronutrient Powders For Fortification of Foods Consumed by Infants and Children (NUTRISUS)) based on the results of a multicenter pragmatic trial following WHO guidelines in settings where the prevalence of anemia in young children is 20% or higher^25^. However, since 2016, political and economic crises in Brazil have drastically reduced access to many primary health programs, which could have contributed to the high prevalence of anemia in young children reported in this study.

Other important actions to prevent and control anemia in young children in Brazil include the promotion of exclusive breastfeeding during the first six months of life and of a healthy and timely introduction of complementary feeding, which should be encouraged in primary healthcare settings. Among children with persistent anemia from one to two years, our results reinforce the importance of promoting continued breastfeeding with nutritious complementary diets and adequate treatment of childhood infections such as malaria. In a previous analysis of the MINA-Brazil cohort, children exposed to gestational malaria and breastfed for at least 12 months had a decreased risk for malaria infection during the first two years of life^26^. Breastfeeding protects young children from infections with the passive transfer of immunoglobulins and other bioactive substances^26^, which could be one possible explanation for the association of prolonged breastfeeding and decreased risk for anemia at age five observed in this study.

To the best of our knowledge, this is the first study to report the significant association between UPF consumption with anemia at age one. A recent systematic review described a higher participation of UPF in children's diet associated with other maternal-child outcomes, such as an increase in weight gain, adiposity measures, overweight, early weaning, lower diet quality, metabolic alterations, diseases, and ingestion of plastic from packaging^27^. As defined by the NOVA classification, UPF are industrial formulations of substances derived from foods with little or no whole food with added colorings, flavorings, emulsifiers, thickeners, and other cosmetic additives to make them palatable or even hyperpalatable^28^. In a previous study, the introduction of UPF in complementary feeding was inversely associated with the duration of continued breastfeeding^29^, providing additional evidence to avoid UPF consumption in childhood.

This study presents some limitations. First, MINA-Brazil cohort participants were not screened for helminthic infections, inherited hemolytic disorders, and other non-nutritional conditions that may be associated with anemia in tropical environments. Accordingly, 4–10% of Amazonian children under the age five tested in population-based cross-sectional surveys harbor one or more soil-transmitted intestinal helminths that may cause or aggravate anemia^22^. However, their contribution to anemia among Amazonian children, although not negligible, seems to be much less pronounced than that in African or Asian populations^22^. Glucose-6-phosphate dehydrogenase (G6PD) deficiency, an X-linked recessive disorder, is the most common inherited cause of hemolytic anemia in the Amazon. It affects 8.3% of the male population of Acre State^30^, who may develop mild to severe hemolysis when exposed to dietary triggers and some medicines, including the locally used antimalarial primaquine. Nevertheless, G6PD deficiency has not emerged as a significant predictor of Hb levels or risk of anemia in Amazonian children^22^. Second, the prevalence of iron deficiency and anemia may have been underestimated in MINA-Brazil cohort participants over time because children lost to follow-up were more likely to live in the poorest households, being more exposed to food insecurity, malaria, and other contributors to nutritional deficiencies and anemia. A range of other conditions (e.g., tropical infections, chronic inflammation, and inherited hemolytic disorders), some of them not addressed by this study, may have contributed to the high prevalence of anemia in Amazonian populations.

## CONCLUSION

Although our study population has access to antenatal care, prompt malaria diagnosis and treatment, and iron and vitamin A supplementation, anemia in MINA-Brazil cohort participants remains common during pregnancy and early childhood, especially at the age one. Moreover, ID and consumption of UPF during age one were associated with elevated risk of anemia. As a multifactorial public health problem, different possible causes should be considered in all efforts and programs to achieve the SDGs targets in this region.
