# Supplementary material for: Prevalence and correlates of childhood anemia in the MINA-Brazil birth cohort study
Source: Rev Saude Publica. 2024 Feb 1;57(Suppl 2):6s. doi: 10.11606/s1518-8787.2023057005637 (PMC10897962; doi:10.11606/s1518-8787.2023057005637)
Supplement: Translation

# Translation: Prevalência e preditores de anemia na infância no estudo de coorte de nascimentos MINA-Brasil

**Authors:** Marly A Cardoso, Bárbara H. Lourenço, Alicia Matijasevich, Marcia C Castro, Marcelo U Ferreira

**Affiliations:** I São Paulo SP Brasil Universidade de São Paulo. Faculdade de Saúde Pública. Departamento de Nutrição. São Paulo, SP, Brasil; II São Paulo SP Brasil Universidade de São Paulo. Faculdade de Medicina. Departamento de Medicina Preventiva. São Paulo, SP, Brasil; III Boston MA United States of America Harvard T.H. Chan School of Public Health. Department of Global Health and Population. Boston, MA, United States of America; IV São Paulo SP Brasil Universidade de São Paulo, Instituto de Ciências Biomédicas. Departamento de Parasitologia. São Paulo, SP, Brasil

**Keywords:** Anemia Ferropriva, Malária, Fatores de Risco, Estudos de Coortes

## Abstract

**OBJETIVO:**

O objetivo deste estudo foi descrever a prevalência e os preditores de anemia na infância em um estudo de coorte de nascimentos de base populacional amazônica.

**MÉTODOS:**

Estimou-se a prevalência de anemia materna no parto (concentração de hemoglobina [Hb] < 110 g/L) em mulheres participantes do estudo de coorte de nascimentos MINA-Brasil e em seus filhos, examinados nas idades um, dois (Hb < 110 g/L) e cinco anos (Hb < 115 g/L). Além disso, as concentrações de ferritina, receptor solúvel de transferrina e proteína C reativa foram medidas em mães no parto e em seus filhos de 1 e 2 anos de idade para estimar a prevalência de deficiência de ferro e sua contribuição para anemia, ajustando para potenciais fatores de confusão por análise de regressão múltipla de Poisson (risco relativo ajustado [RR_a_]).

**RESULTADOS:**

As prevalências com intervalo de confiança (IC) de 95% de anemia materna, deficiência de ferro e anemia ferropriva no parto foram de 17,3% (14,0–21,0%), 42,6% (38,0–47,2%) e 8,7% (6,3–11,6%), respectivamente (n = 462). No primeiro ano de idade (n = 646), 42,2% (38,7–45,8%) das crianças estudadas eram anêmicas, 38,4% (34,6–42,3%) eram deficientes em ferro e 26,3 (23,0–29,9%) tinham anemia ferropriva. Aos dois anos de idade (n = 761), esses valores diminuíram para 12,8% (10,6–15,2%), 18,1% (15,5–21,1%) e 4,1% (2,8–5,7%), respectivamente; aos cinco anos de idade (n = 655), 5,2% (3,6–7,2%) eram anêmicos. A deficiência de ferro (RR_a_ = 2,19, IC95%: 1,84–2,60) e consumo de alimentos ultraprocessados (AUP) (RR_a_ = 1,56, IC95%: 1,14–2,13) foram contribuintes significantes para anemia no 1° ano de idade, após ajuste para escolaridade materna. Aos 2 anos, a anemia associou-se significativamente à anemia materna no parto (RR_a_ = 1,67; IC95%: 1,17–2,39), malária desde o nascimento (2,25; 1,30–3,87) e deficiência de ferro (2,15; 1,47–3,15), após ajuste para idade das crianças e índice de riqueza familiar.

**CONCLUSÕES:**

A anemia continua sendo altamente prevalente durante a gravidez e a primeira infância na Amazônia. Políticas públicas de saúde devem abordar a deficiência de ferro, o consumo de AUP, a anemia materna e a malária para prevenir e tratar a anemia em crianças amazônicas.

## INTRODUÇÃO

Estima-se que quase um terço da população mundial seja anêmica, com aumento da morbidade e mortalidade, diminuição da produtividade no trabalho e comprometimento do neurodesenvolvimento infantil^1^. Lactentes, crianças pequenas e gestantes estão entre as populações mais vulneráveis à anemia no mundo^2^. No período de 2000 a 2019, a prevalência de anemia em crianças de 6 a 59 meses diminuiu de 48% para 40% em todo o mundo e de 30% para 20% na América Latina e no Caribe; no entanto, a carga da anemia infantil permanece impressionante em muitos países de baixa e média renda^3^. Meta-análises recentes revelam disparidades regionais na prevalência de anemia infantil no Brasil que estão escondidas em estimativas gerais do país^4,5^. Por exemplo, a Pesquisa Nacional de Nutrição Infantil (ENANI-2019) estimou que 10% dos pré-escolares residentes em áreas metropolitanas brasileiras eram anêmicos; no entanto, na região Norte – região que compreende a maior parte da Bacia Amazônica do Brasil –, a prevalência chegou a 17%^6^. As estimativas correspondentes para crianças de 6 a 23 meses foram de 20% em todo o país e 30% no Norte^6^.

A deficiência de ferro é responsável por cerca de 50% da carga global da anemia na infância^7,8^. Para a prevenção primária da anemia ferropriva em crianças pequenas, a Organização Mundial da Saúde (OMS) recomenda o aleitamento materno exclusivo (AME) por seis meses e o aleitamento materno contínuo (AM) até os 2 anos ou mais, além de alimentação complementar adequada com suplementos de ferro ou múltiplos micronutrientes^9^. No entanto, os programas de suplementação para prevenção da anemia têm uma cobertura relativamente baixa na maioria dos países de baixa e média renda^7^ e falham em abordar causas não nutricionais de anemia infantil, como infecções helmínticas transmitidas pelo solo, esquistossomose e malária^2^.

Este estudo teve como foco a anemia na infância na região Norte do Brasil, região com a maior prevalência de anemia em pré-escolares estimada na mais recente pesquisa nacional^6^. Descrevemos a prevalência de anemia nos primeiros cinco anos de acompanhamento de crianças participantes de um estudo de coorte de nascimentos de base populacional em andamento na Amazônia e identificamos preditores independentes de risco de anemia que podem constituir alvos potenciais para intervenções de saúde pública.

## MÉTODOS

### Desenho do Estudo, População e Coleta de Dados

O estudo de coorte de nascimentos Saúde e Nutrição Materno-Infantil no Acre (MINA-Brasil) teve início em 2015 com 1.246 pares mãe-filho na cidade de Cruzeiro do Sul (CZS), na Amazônia Ocidental, Estado do Acre. Objetivou-se caracterizar o efeito de uma ampla gama de exposições precoces sobre a saúde infantil^10^. A taxa de mortalidade infantil em CZS foi estimada em 10,8 óbitos por 1.000 nascidos vivos em 2015 e 10,6 por 1.000 em 2020^11^. CZS experimenta transmissão de malária durante todo o ano, com quase 90% de todas as infecções por malária em crianças pequenas relacionadas ao *Plasmodium vivax*^12^. A incidência anual de malária (IPA; número de casos confirmados laboratorialmente por 1.000 pessoas por ano) foi estimada em 231,9 em CZS em 2016^12^, uma das mais altas entre os municípios brasileiros. As duplas mãe-bebê foram cadastradas na gestação em clínicas públicas de pré-natal ou no parto no Hospital da Mulher e da Criança do Vale do Juruá, única maternidade de CZS. O termo de consentimento livre e esclarecido foi obtido no momento da inclusão das mães ou cuidadores (no caso de mães adolescentes). Os protocolos de pesquisa foram aprovados pelo Comitê de Ética em Pesquisa da Faculdade de Saúde Pública da Universidade de São Paulo, Brasil (# 872.613, 2014; # 2.358.129, 2017).

Durante a gestação, avaliações clínicas e laboratoriais foram realizadas pela equipe de pesquisa em uma subamostra (n = 557) de toda a coorte (n = 1.246). No momento do parto, foram obtidos os seguintes dados de entrevistas e prontuários: sociodemográficos (escolaridade materna, cor da pele autorreferida das mães, se a família era apoiada pelo Programa Bolsa Família, programa de transferência condicionada de renda, e índice de riqueza baseado em bens usado como indicador de nível socioeconômico) e dados maternos e perinatais (como paridade, número de consultas de pré-natal, tabagismo e etilismo durante a gestação, concentração de hemoglobina materna e peso corporal no parto, idade gestacional e tipo de parto, sexo da criança e peso ao nascer). A partir do cartão de pré-natal, foram coletadas informações sobre a altura materna (m) e o peso pré-gestacional (kg) de todas as participantes. O índice de massa corporal (IMC) pré-gestacional foi categorizado em baixo peso (< 18,5 kg/m^2^), peso normal (18,5 a 24,9 kg/m^2^), sobrepeso (25,0 a 29,9 kg/m^2^) ou obesidade (≥ 30,0 kg/m^2^), conforme definido pela OMS. A diferença entre o peso no parto e o peso pré-gestacional foi utilizada para estimar o ganho de peso gestacional (GPG) materno. Com base nas categorias de IMC pré-gestacional, o GPG foi classificado como insuficiente, adequado ou excessivo, seguindo as diretrizes de 2009 do Instituto de Medicina^13^.

Após o nascimento, as avaliações clínicas com coleta de sangue foram realizadas nas unidades de saúde quando as crianças tinham um, dois e cinco anos de idade, conforme descrito anteriormente^10^. As medidas antropométricas foram realizadas em duplicata, utilizando-se procedimentos padronizados. Os índices antropométricos das crianças em escores-z foram calculados segundo idade e sexo, seguindo os Padrões de Crescimento Infantil da OMS; déficit estatural e sobrepeso foram definidos como escore-z de comprimento ou estatura para idade < −2 e escore-z de índice de massa corporal (IMC) para idade > 2, respectivamente^14^.

Em cada consulta de acompanhamento infantil, questionários estruturados foram aplicados às mães ou responsáveis para atualização dos dados, incluindo práticas alimentares infantis, morbidades desde o nascimento e outras características. As mães relataram se a criança estava sendo amamentada (sim ou não) e, em caso negativo, a idade do desmame. Foram consideradas em aleitamento materno exclusivo as crianças que receberam leite materno sem outros alimentos ou bebidas, exceto medicamentos prescritos, soluções de reidratação oral, vitaminas e minerais. O aleitamento materno continuado (AM) foi estimado em dias e classificado como AM prolongado quando ≥ 365 dias. Nas visitas de acompanhamento de 1 e 2 anos, as informações sobre alimentação complementar foram avaliadas com um questionário estruturado de frequência alimentar sobre o consumo de alimentos e bebidas no dia anterior, conforme detalhado em outro estudo^15^. Foram incluídos no questionário os seguintes alimentos ultraprocessados (AUP): iogurte industrializado, suco artificial de frutas, refrigerantes, balas, biscoitos, salgadinhos embalados, cachorro-quente e macarrão instantâneo e “outros AUP” (achocolatados, sorvetes, geleias, bolos e sopas industrializadas). Em seguida, a prevalência de consumo de AUP foi estimada a partir do consumo de pelo menos um alimento da categoria no dia anterior.

Amostras de sangue venoso (10 mL para gestantes do terceiro trimestre e 5 mL para crianças) foram coletadas para mensuração de indicadores nutricionais bioquímicos durante a gestação e nos acompanhamentos de 1 e 2 anos; no seguimento de 5 anos, apenas a concentração de hemoglobina (Hb) sanguínea foi medida. A concentração de Hb < 110 g/L, medida em clínicas de pré-natal utilizando um Hemocue portátil (Hb301; Angelholm, Suécia) definiu anemia na gestação^16^. Um contador de células SDH-20 Labtest (Labtest, Lagoa Santa, Brasil) foi usado para medir os níveis de Hb das mães na maternidade. Um contador de células ABX Micro 60 (Horiba, Montpellier, França) foi usado para medir as concentrações de Hb das crianças nas visitas de acompanhamento de 1 e 2 anos; no seguimento de 5 anos, foi utilizado hemoglobinômetro portátil da marca Hemocue. As amostras de sangue foram protegidas da luz e centrifugadas até duas horas após a coleta; amostras de soro e plasma foram congeladas a −20°C, enviadas em gelo seco para São Paulo e mantidas a −70°C até novas análises. As concentrações plasmáticas de ferritina e receptor solúvel de transferrina foram medidas por ensaio imunoenzimático (Ramco, Houston, TX, EUA). A cromatografia líquida de alta performance (HPLC) foi utilizada para medir as concentrações séricas de retinol, conforme descrito anteriormente^17^. Fluoroimunoensaio (PerkinElmer; Wallac Oy, Turku, Finlândia) foi utilizado para medir as concentrações séricas de folato. Um Sistema de Imunoquímica IMMAGE (Beckman Coulter, Brea, CA, EUA) foi usado para determinar a proteína C reativa. As análises foram submetidas ao controle de qualidade interno e externo com uso rotineiro de amostras cegas para cada ensaio, com coeficientes de variação < 7%.

Os pontos de corte estabelecidos pela OMS foram adotados para definir deficiências vitamínicas: concentrações séricas de retinol < 0,7 μmol/L para deficiência de vitamina A (DVA) e < 1,05 μmol/L para insuficiência de vitamina A (IVA)^18^,e concentração sérica de folato < 3 ng/mL (gestantes) ou < 4 ng/mL (crianças) para deficiência de folato^19^. A deficiência de ferro (DF) foi definida como concentração plasmática de ferritina < 12 μg/L (crianças) ou < 15 μg/L (gestantes), combinada com uma concentração de receptor solúvel de transferrina > 8,3 mg/L, ou ferritina plasmática < 30 μg/L combinada com uma concentração de proteína C reativa ≥ 5 mg/L (sugestiva de inflamação aguda)^20^.

O diagnóstico de malária durante a gestação foi feito por microscopia durante visitas às unidades de saúde e/ou por reação em cadeia da polimerase (PCR) em tempo real no parto^21^. A malária em crianças pequenas foi diagnosticada por microscopia durante visitas às unidades de saúde. As notificações de casos durante a gestação e os dois primeiros anos de vida foram recuperadas do banco de dados eletrônico do Ministério da Saúde do Brasil (SIVEP Malária), utilizando-se uma estratégia de relacionamento previamente descrita^12^.

Anemia, deficiências nutricionais e malária diagnosticadas durante as consultas de acompanhamento foram tratadas pelos médicos da equipe de pesquisa. Às crianças com anemia foi prescrito sulfato ferroso (dose: 3–6 mg de ferro elementar/kg por dia até 60 mg/dia durante quatro meses) para o tratamento presuntivo da deficiência de ferro, mas a adesão a esse tratamento não foi avaliada.

### Medidas dos Principais Desfechos

Anemia na infância foi definida como Hb < 110 g/L para crianças < 5 anos. Na consulta de acompanhamento de 5 anos, a Hb < 115 g/L definiu anemia para crianças ≥ 5 anos. A anemia grave foi classificada como Hb < 70 g/L e < 80 g/L para crianças < 5 e ≥ 5 anos, respectivamente^16^.

### Análise de dados

As características maternas e da criança foram descritas por meio de frequências absolutas e proporções (%) com intervalos de confiança (IC) de 95%. Foram calculadas a média e o DP para a idade e os valores medianos e intervalos interquartis (IIQ, percentis 25 e 75) para os indicadores nutricionais bioquímicos. Os testes χ^2^ de Pearson ou exato de Fisher foram utilizados para comparar proporções.

Os riscos relativos (RR) com IC95% foram estimados por meio de modelos múltiplos de regressão de Poisson com variância robusta para identificar fatores associados à anemia infantil em cada consulta de seguimento e à “anemia persistente” (crianças anêmicas tanto no 1° quanto no 2° ano de vida). Coeficientes de regressão linear múltipla (β) também foram calculados para descrever os preditores das concentrações de Hb (variável dependente) em cada avaliação de acompanhamento. As variáveis associadas aos desfechos em nível de significância < 20% na análise bruta foram inseridas em modelos de regressão múltipla. Utilizou-se uma abordagem hierárquica baseada em marcos conceituais^7,22^ para selecionar determinantes distais (fatores demográficos e socioeconômicos), subjacentes (cuidados pré-natais, características obstétricas e do nascimento) e imediatos (práticas alimentares infantis, estado nutricional e morbidades) da anemia infantil nos modelos finais ajustados (Figura 1). Em cada nível de determinação, as covariáveis foram mantidas no modelo se estivessem associadas ao desfecho com p < 0,10 e/ou para variáveis ordinais com mais de duas categorias se seguissem um padrão dose-resposta. Os valores faltantes nas covariáveis categóricas foram mantidos no modelo criando uma nova categoria de valor faltante. Todos os valores de p relatados são bicaudais e o nível de significância foi estabelecido em p < 0,05. As análises estatísticas foram realizadas no programa Stata versão 15.0 (StataCorp, College Station, TX, EUA).

**Figura 1:** Abordagem hierárquica causal da anemia na infância baseada nas variáveis disponíveis no estudo de coorte de nascimentos MINA-Brasil, adaptado de marcos conceituais anteriores^7,22^

## RESULTADOS

Na linha de base, obtivemos dados de 1.246 participantes da coorte MINA Brasil. Após a exclusão de 22 gêmeos, um total de 1.224 pares mãe-filho foram elegíveis para esta análise. Dessas, 79 faltaram a todas as consultas de acompanhamento pós-natal e seis crianças morreram até os cinco anos de idade (Figura 2). As crianças que participaram desde o nascimento até diferentes acompanhamentos e aquelas que deixaram de ser acompanhadas antes do acompanhamento de 5 anos (n = 514) apresentaram características perinatais similares quanto ao sexo, idade gestacional, prematuridade e peso ao nascer, mas diferiram significantemente na proporção de crianças de famílias mais pobres (36,2% *versus* 44,1%) e de mães com ≤ 9 anos de estudo (26,4% *versus* 47,5%), respectivamente, com p < 0,01 (c^2^ teste) para ambos.

**Figura 2:** Fluxograma do estudo de coorte de nascimentos MINA-Brasil desde a gestação até as consultas de acompanhamento de 5 anos.

A prevalência geral de anemia na infância diminuiu de 42% para 13% e 5% nas avaliações de 1, 2 e 5 anos, respectivamente. Foram observados dois casos (0,3%) de anemia grave (Hb < 70 g/L) no acompanhamento de 1 ano; a prevalência de anemia moderada foi de 14,2%, 1,5% e 1,5% aos 1, 2 e 5 anos de idade, respectivamente (Tabela 1). Na análise bruta, o menor índice de riqueza, a menor idade materna, a menor escolaridade materna, o aleitamento materno prolongado e o consumo de AUP associaram-se significantemente à anemia no primeiro ano de idade. Aos dois anos, crianças de famílias com menor índice de riqueza, filhas de mãe multípara, com < 6 consultas de pré-natal e história de uma ou mais infecções por malária desde o nascimento apresentaram maior risco de anemia. Aos cinco anos, apenas o aleitamento materno prolongado associou-se significantemente com menor risco de anemia.

**Table 1:** Características dos participantes da coorte de nascimentos MINA-Brasil em relação à anemia infantil nas consultas de acompanhamento.

Característica			Consultas de acompanhamento da criança						
			Totais^a^ (%)	Anemia^b^ no 1° ano (n = 768)		Anemia^b^ no 2° ano (n = 846)		Anemia no 5° ano (n = 655)	
				n (%)	valor de p	n (%)	valor de p	n (%)	valor de p
Geral			1.224	324 (42,2)	-	108 (12,8)	-	31 (5,2)	-
Características basais									
	Idade materna no parto (anos)				0,009		0,893		0,832
		< 19	229 (18,7)	65 (52,9)		16 (12,4)		6 (5,6)	
		≥ 19	995 (81,3)	259 (40,2)		92 (12,8)		28 (5,1)	
	Cor da pele materna autorreferida				0,913		0,559		0,233
		Branca	149 (12,5)	39 (41,9)		12 (10,9)		2 (2,5)	
		Preta, parda, indígena e amarela	1.042 (87,5)	282 (42,5)		93 (12,9)		32 (5,7)	
	Índice de riqueza em tercis				< 0,001		0,004		0,768
		Menor	400 (33,6)	113 (52,1)		43 (18,8)		9 (5,8)	
		Segundo	392 (32,9)	121 (46,5)		31 (10,8)		10 (4,4)	
		Maior	399 (33,5)	87 (31,2)		31 (9,8)		15 (5,8)	
	Escolaridade materna (anos)				< 0,001		0,181		0,647
		≤ 9	422 (35,5)	123 (54,7)		36 (15,1)		10 (6,0)	
		> 9	768 (64,5)	197 (37,2)		69 (11,7)		24 (5,1)	
	Programa Bolsa Família				0,032		0,064		0,233
		Não	720 (60,5)	188 (39,5)		57 (11,0)		23 (5,6)	
		Sim	471 (39,6)	133 (47,5)		48 (15.,4)		11 (4,7)	
	Consultas de pré-natal				0,185		0,047		0,455
		< 6	288 (23,7)	66 (47,1)		29 (17,5)		4 (3,7)	
		≥ 6	926 (76,3)	256 (41,0)		79 (11,7)		30 (5,5)	
	Malária durante a gravidez				0,358		0,909		0,354
		Não	1.142 (93,3)	298 (41,7)		101 (12,8)		31 (5,0)	
		Sim	82 (6,7)	26 (48,2)		7 (12,3)		3 (8,6)	
	Ganho de peso gestacional^c^				0,434		0,760		0,688
		Insuficiente	341 (31,3)	93 (44,1)		27 (11,3)		7 (3,9)	
		Adequado	392 (36,0)	101 (38,7)		35 (12,4)		12 (5,5)	
		Excessivo	357 (32,8)	110 (43,1)		37 (13,4)		12 (5,6)	
	Paridade				0,159		0,034		0,200
		Primíparas	498 (41,8)	126 (39,5)		33 (9,7)		16 (4,3)	
		Multíparas	693 (58,2)	195 (44,6)		72 (44,7)		18 (6,6)	
	Tipo de parto				0,971		0,432		0,570
		Vaginal	660 (53,9)	169 (42,3)		61 (13,6)		19 (5,7)	
		Cesariana	564 (46,1)	155 (42,1)		47 (11,8)		15 (4,7)	
	Prematuridade (idade gestacional) (semanas)				0,148		0,310		0,670
		Não (≥ 37)	1.120 (91,5)	304 (42,9)		102 (13,1)		32 (5,3)	
		Sim (< 37)	104 (8,5)	20 (33,3)		6 (8,8)		2 (3,9)	
	Peso ao nascer (gramas)				0,486		0,485		0,706
		2.500 – < 4.000	1.061 (86,8)	288 (42,9)		94 (12,9)		28 (4,9)	
		< 2.500	86 (7,0)	18 (36,7)		5 (8,6)		3 (7,3)	
		≥ 4.000	76 (6,2)	17 (36,2)		9 (16,1)		3 (6,8)	
Características da criança durante o acompanhamento									
	Aleitamento materno exclusivo ≥ 90 dias				0,760		0,295		0,080
		Não	691 (67,0)	198 (41,7)		68 (13,7)		25 (6,5)	
		Sim	340 (33,0)	125 (42,8)		30 (11,1)		7 (3,2)	
	Aleitamento materno prolongado (≥ 365 dias)				< 0,001		0,394		0,039
		Não	318 (34,9)	63 (28,3)		29 (11,3)		15 (7,8)	
		Sim	593 (65,1)	216 (46,2)		79 (13,4)		16 (3,9)	
	Malária nos primeiros 2 anos				0,338		0,016		0,386
		Não	808 (94,6)	313 (41,9)		97 (12,1)		28 (5,0)	
		Sim	46 (5,4)	11 (52,4)		11 (24,4)		2 (9,1)	
	Consumo de feijão^d^				0,362		0,056		0,468
		Não	-	147 (42,6)		69 (14,9)		14 (4,4)	
		Sim	-	118 (39,1)		39 (10,4)		15 (5,7)	
	Consumo de carnes^d^				0,686		0,170		0,473
		Não	-	77 (41,0)		17 (17,2)		2 (3,2)	
		Sim	-	246 (42,6)		91 (12,3)		28 (5,4)	
	Consumo de alimentos ultraprocessados^d^,^e^				0,007		0,746		0,531
		Não	-	29 (29,6)		7 (11,5)		1 (2,9)	
		Sim	-	295 (44,0)		101 (12,9)		29 (5,3)	

a Os totais diferem devido à falta de valores; Valores de p para o teste χ^2^ de Pearson ou teste exato de Fisher (contagem de células < 5).

b Critérios de classificação da Organização Mundial da Saúde para anemia (2011): concentrações sanguíneas de hemoglobina < 110 g/L para crianças menores de 5 anos e < 115 g/L para crianças ≥ 5 anos.

c Diretrizes do *Institute of Medicine*, 2009.

d Frequência alimentar de consumo no dia anterior.

e Consumo de pelo menos um alimento ultraprocessado.

A Tabela 2 mostra os valores médios de idade (DP), frequências de uso de suplementos de ferro e/ou vitaminas, prevalência (IC95%) de deficiências nutricionais e intervalos interquartis (IIQ) dos parâmetros bioquímicos nas diferentes ondas de toda a coorte. Entre gestantes e parturientes, os suplementos mais utilizados foram ferro (30–60 mg/dia) e ácido fólico (5 mg/dia), seguindo as diretrizes nacionais de assistência pré-natal. No momento do parto, foram observadas frequências similares (em torno de 30%) de baixo peso e GPG excessivo. A anemia foi comum entre gestantes (17%) e parturientes (38%). Inadequações do estado nutricional de ferro ou vitamina A foram frequentes entre gestantes e crianças menores de dois anos; deficiência de ácido fólico foi incomum, com prevalência < 1% em mães e crianças. As deficiências combinadas de ferro e vitamina A foram mais comuns aos dois anos de idade (6,7%).

**Table 2:** Características nutricionais dos participantes da coorte MINA-Brasil (n = 1.224).

Característica						Consultas de acompanhamento da criança					
		Gestantes		Parturientes		1 ano		2 anos		5 anos	
		n = 557	Média (DP)	n = 1.224	Média (DP)	n = 774	Média (DP) ou %	n = 854	Média (DP) ou %	n = 682	Média (DP) ou %
Média de idade (anos ou meses)			24,8 anos (6,5)		24,9 anos (6,6)		12,7 meses (0,7)		23,8 meses (1,4)		63,4 meses (2,7)
Sexo feminino			-		-	404	52,2	427	50,0	336	49,3
Uso de suplementação vitamínica e/ou mineral		n = 557	%	n = 1.224	%	n = 774	%	n = 854	%	n = 682	%
	Ferro		69,1		65,9		9,4		3,5		0,4
	Multinutrientes		38,1		42,6		32,7		26,4		27,1
	Ácido fólico (5 mg)		70,7		66,4		-		-		-
	Vitamina A		34,3		61,2		11,2		31,4		21,0
Prevalência de anemia e deficiências de micronutrientes		n^a^	% (IC95%)	N	% (IC95%)	n	% (IC95%)	n	% (IC95%)	n	% (IC95%)
	Anemia^b^	469	17,3 (14,0–21,0)	1.156	37,7 (35.0–40.5)	768	42,2 (38,7–45,8)	846	12,8 (10,6–15,2)	655	5,2 (3,6–7,2)
	Deficiência de ferro^c^	463	42,6 (38,0–47,2)		-	646	38,4 (34,6–42,3)	761	18,1 (15,5–21,1)		-
	Anemia ferropriva	462	8,7 (6,3–11,6)		-	646	26,3 (23,0–29,9)	761	4,1 (2,8–5,7)		-
	Deficiência de vitamina A^d^	467	6,4 (4,4–9,0)		-	533	1,7 (0,8–3,2)	703	24,8 (21,6–28,1)		-
	Insuficiência de vitamina A^d^	467	19,7 (16,2–23,6)		-	533	10,3 (7,9–13,2)	703	41,7 (38,0–45,4)		-
	Deficiência de ácido fólico^e^	464	0,7 (0,1–1,9)		-	484	1,0 (0,3–2,4)	490	0,4 (0,1–1,50)		
	Deficiências combinadas de ferro e vitamina A	461	2,8 (1,5–4,8)		-	532	0,8 (0,2–1,9)	699	6,7 (5,0–8,8)		-
	Proteína C reativa ≥ 5 mg/L (ponto de corte para inflamação aguda)	465	0,35 (0,31–0,40)			641	12,9 (10,4–15,8)	712	10,5 (8,4–13,0)		-
Parâmetro bioquímico		n	Mediana (IIQ)	N	Mediana (IIQ)	n	Mediana (IIQ)	n	Mediana (IIQ)	n	Mediana (IIQ)
	Hemoglobina (g/L)	469	119 (112–125)	1.156	113 (104–120)	768	112 (104–119)	846	121 (114–127)	655	128 (122–133)
	Ferritina plasmática (μg/L)	463	17,0 (10,0–27,0)		-	639	21,0 (12,0–33,0)	725	31,0 (21,0–43,0)		-
	Retinol sérico (μmol/L)	467	1,9 (1,2–2,7)		-	533	1,9 (1,4–2,8)	703	1,2 (0,7–1,7)		-
	Ácido fólico sérico (ng/mL)	464	9,6 (7,1–13,4)		-	484	14,0 (11,0–18,2)	490	14,3 (11,3–18,8)		-

IC95%: intervalo de confiança de 95%; DP: desvio padrão; IIQ: intervalos interquartis.

a Amostras de sangue no terceiro trimestre gestacional (idade gestacional média: 27,8, DP: 1,6, variando de 24 a 34 semanas).

b Critérios de classificação da OMS para anemia (2011): concentrações de hemoglobina < 110 g/L para gestantes e crianças menores de 5 anos, e < 115 g/L para crianças ≥ 5 anos.

c Deficiência de ferro: gestantes: ferritina < 15 μg/L; crianças: ferritina < 12 μg/L e receptor solúvel de transferrina > 8,3 mg/L.

d Deficiência de vitamina A < 0,7 μmol/L, insuficiência de vitamina A < 1,05 μmol/L.

e Ponto de corte de ácido fólico: gestantes < 3,0 ng/mL; crianças < 4,0 ng/mL.

A DF (RR ajustado [RR_a_] = 2,19, IC95%: 1,84–2,60) e o consumo de AUP (RR_a_ = 1,56, IC95%: 1,14–2,13) associaram-se a risco elevado de anemia no primeiro ano de vida, após ajuste para escolaridade materna na análise de regressão com ajuste múltiplo (Tabela 3). No acompanhamento de 2 anos, a anemia materna no parto (RR_a_ = 1,67, IC95%: 1,17–2,39), malária nos primeiros 2 anos de vida (RR_a_ = 2,25, IC95%: 1,30–3,87) e DF (RR_a_ = 2,15, IC95%: 1,47–3,15) foram significantemente associados ao aumento do risco de anemia, após ajuste para idade da criança e índice de riqueza familiar. O risco de anemia persistente nas consultas de acompanhamento de 1 e 2 anos (n = 53) associou-se positivamente com o sexo masculino (RR_a_ = 1,87, IC95%: 1,05–3,31), malária nos dois primeiros anos de vida (RR_a_ = 4,30, IC95%: 2,33–7,94), aleitamento materno prolongado (RR_a_ = 2,48, IC95%: 1,11–5,51) e insuficiência de vitamina A (RR_a_ = 2,03, IC95%: 1.16–3.54); o consumo de carnes emergiu como fator protetor (RR_a_ = 0,52, IC95%: 0,29–0,95). Aos 5 anos, apenas a anemia materna no parto associou-se positivamente com o risco de anemia (RR_a_ = 2,32, IC95%: 1,11– 4,84), enquanto o aleitamento materno prolongado associou-se inversamente ao risco de anemia (RR_a_ = 0,45, IC95%: 0,22–0,94).

**Table 3:** Risco relativo (RR_a_) para anemia infantil e coeficientes de regressão ajustados (aβ) para preditores de valores de hemoglobina (Hb, g/L) no estudo de coorte MINA-Brasil.

Exposição		Risco de anemia no acompanhamento de 1 ano (anêmicos/total = 324/768)		Risco de anemia no acompanhamento de 2 anos (n = 846)				Risco de anemia no acompanhamento de 5 anos (anêmicos/total = 34/655)^a^	
				Todas as crianças com anemia n = 108		Crianças com anemia persistente de 1 ano a 2 anos (n = 53)			
		RR_a_ (IC95%)	valor de p	RR_a_ (IC95%)	valor de p	RR_a_ (IC95%)	valor de p	RR_a_ (IC95%)	valor de p
Índice de riqueza (tercis)									
	Menor	-	-	1		-	-	-	-
	Segundo	-	-	0,61 (0,39 a 0,95)	0,030	-	-	-	-
	Maior	-	-	0,96 (0,39 a 2,36)	0,029	-	-	-	-
Escolaridade materna (anos)									
	≤ 9	1		-	-	-	-	-	-
	10–12	0,83 (0,71 a 0,99)	0,034	-	-	-	-	-	-
	> 12	0,70 (0,53 a 0,91)	0,008	-	-	-	-	-	-
Idade da criança (meses)		-	-	1,18 (1,06 a 1,32)	0,002	-	-	-	-
Criança do sexo masculino		-	-	-	-	1,87 (1,05 a 3,31)	0,047	-	-
Anemia materna no parto		-	-	1,67 (1,17 a 2,39)	0,005	-	-	2,32 (1,11 a 4,84)	0,025
Aleitamento materno prolongado (≥ 365 dias)		-	-	-	-	2,48 (1,11 a 5,51)	0,001	0,45 (0,22 a 0,94)	0,032
Ingestão de AUP no 1° ano		1,56 (1,14 a 2,13)	0,005	-	-	-	-	-	-
Consumo alimentar de carnes		-	-	-	-	0,52 (0,29 a 0,95)	0,026		
Malária nos primeiros 2 anos		-	-	2,25 (1,30 a 3,87)	0,003	4,38 (2,33 a 7,94)	< 0,001	-	-
Deficiência de ferro		2,19 (1,84 a 2,60)	< 0,001	2,15 (1,47 a 3,15)	< 0,001	-	-	-	-
Insuficiência de vitamina A		-	-	-	-	2,03 (1,16 a 3,54)	0,013	-	-
		**Preditores de Hb no acompanhamento de 1 ano**		**Preditores de Hb no acompanhamento de 2 anos**				**Preditores de Hb no acompanhamento de 5 anos**	
		**(R^2^ = 0,200)**		**(R^2^ = 0,095)**				**(R^2^ = 0,061)**	
		**aβ (IC95%)**	**valor de p**	**aβ (IC95%)**	**valor de p**			**aβ (IC95%)**	**valor de p**
Índice de riqueza (tercis)									
	Menor	-	-	Ref.			-		-
	Segundo	-	-	1,86 (0,13 a 3,60)	0,036		-	-	-
	Maior	-	-	2,68 (0,95 a 4,41)	0,002		-	-	-
Escolaridade materna (anos)									
	≤ 9	Ref.		-	-			-	-
	10–12	2,82 (0,74 a 4,91)	0,008	-	-		-	-	-
	> 12	3,84 (1,13 a 6,55)	0,006	-	-		-	-	-
Mãe primípara		-	-	1,92 (0,50 a 3,33)	0,008		-	-	-
Anemia materna no parto		-	-	−3,12 (−4,54 a −1,71)	< 0,001		-	−3,16 (−4,80 a −1,52)	< 0,001
Aleitamento materno prolongado (≥ 365 dias)		-	-	-	-		-	2,16 (0,39 a 3,93)	0,017
Malária nos primeiros 2 anos		-	-	−4,15 (−7,25 a −1,05)	0,009		-	−9,26 (−13,56 a 4,97)	< 0,001
Consumo de AUP no 1° ano		-	-	-	-		-	−2,47 (−4,94 a −0,01)	0,050
Consumo do grupo feijão aos 2 anos		-	-	1,90 (0,51 a 3,30)	0,007		-	-	-
Deficiência de ferro		−9,55 (−11,44 a −7,67)	< 0,001	−4,18 (−5,96 a −2,40)	< 0,001		-	-	-
Insuficiência de vitamina A		−3,26 (−6,25 a −0,27)	0,033	-	-		-	-	-

AUP: alimentos ultraprocessados.

a Das 34 crianças com anemia, os casos anêmicos persistentes de 1 a 5 anos foram n = 8.

Preditores de concentrações de Hb na infância também foram explorados (Tabela 3). No primeiro ano de vida, a escolaridade materna associou-se positivamente com a concentração de Hb, enquanto a DF e a IVA associaram-se negativamente. Aos dois anos, filhos de famílias do estrato de maior riqueza, filhos de mães primíparas e que consumiam feijão, apresentaram maiores concentrações de Hb, enquanto a anemia materna no parto, malária nos primeiros 2 anos e deficiência de ferro associaram-se negativamente à concentração de Hb. Aos cinco anos, o aleitamento materno prolongado associou-se positivamente à concentração de Hb; por outro lado, anemia materna, consumo de AUP no primeiro ano e malária nos dois primeiros anos de vida apresentaram associação negativa.

## DISCUSSÃO

Neste estudo, abordamos algumas das causas mais prováveis de anemia infantil na Amazônia. Mostramos que a deficiência de ferro (DF) ainda é muito comum, afetando 43% das gestantes locais e 38% das crianças com um ano de idade, mas sua contribuição para a anemia parece variar com a idade. Assim, diagnosticamos DF em 51% das gestantes e 62% das crianças de um ano com anemia, o que foi observado em apenas 32% das crianças anêmicas aos dois anos. DVA e IVA também foram comuns durante a gestação (6% e 20%, respectivamente) e em crianças de dois anos (25% e 42%, respectivamente), o que foi substancialmente menos frequente em crianças de um ano de idade (2% e 10%, respectivamente), nas quais a anemia infantil é mais prevalente. As evidências de que a IVA ou DVA contribuem para a anemia em nossa população são duas: encontramos uma associação significante entre IVA e risco elevado de anemia persistente na primeira infância, bem como um impacto negativo da IVA nos níveis de Hb no primeiro ano de vida.

Crianças nascidas de mães anêmicas no momento do parto apresentaram maior probabilidade de serem anêmicas aos dois e cinco anos de idade, após ajuste para potenciais fatores de confusão. Embora essa associação seja provavelmente causal, confundimento residual também deve ser considerado, uma vez que pares mãe-filho que compartilham o mesmo domicílio estão igualmente expostos à pobreza, insegurança alimentar e outros contribuintes-chave para deficiências nutricionais e anemia. Destacamos que evidências recentes mostram que a anemia na gestação aumenta o risco de DF e anemia em lactentes, sugerindo que os benefícios da prevenção da anemia na gestação podem se estender à prole, reduzindo o risco de anemia na primeira infância^23^. Nossos resultados reforçam o argumento da suplementação rotineira de ferro na gestação, preconizado pela OMS em áreas com prevalência > 40%^16^.

A deficiência de folato foi rara entre os participantes da coorte MINA-Brasil, provavelmente devido à fortificação com ácido fólico da farinha de trigo implementada no Brasil em 2004. O Brasil adotou a fortificação das farinhas de trigo e milho com 4,2 mg de ferro e 150 mg de ácido fólico adicionados por cada 100 g. No entanto, a fortificação com ferro das farinhas parece ser pouco efetiva na prevenção da anemia parece em crianças pequenas^24^. A transição para alimentos complementares, à medida que as crianças passam a consumir dietas domiciliares, está associada à ingestão insuficiente de ferro, combinada com a baixa ingestão dietética de outros micronutrientes^7^. Assim, desde 2011, o uso de múltiplos micronutrientes em pó (MNP) tem sido recomendado pela OMS como estratégia domiciliar para prevenir e controlar a anemia infantil. Em 2014, o Ministério da Saúde do Brasil lançou a Estratégia de Fortificação da Alimentação Infantil com Micronutrientes em Pó (NutriSUS) com base nos resultados de um ensaio pragmático multicêntrico seguindo as diretrizes da OMS em ambientes onde a prevalência de anemia em crianças pequenas é de 20% ou mais^25^. No entanto, desde 2016, as crises políticas e econômicas no Brasil reduziram drasticamente o acesso a muitos programas da atenção primária à saúde, o que pode ter contribuído para a alta prevalência de anemia em crianças pequenas relatada neste estudo.

Outras ações importantes para a prevenção e controle da anemia em crianças pequenas no Brasil incluem a promoção do aleitamento materno exclusivo nos primeiros seis meses de vida e a introdução saudável e oportuna da alimentação complementar, que deve ser incentivada na atenção primária à saúde. Entre as crianças com anemia persistente entre o primeiro e o segundo ano de vida, nossos resultados reforçam a importância da promoção do aleitamento materno contínuo com dietas complementares nutritivas e tratamento adequado de infecções infantis, como a malária. Em uma análise anterior da coorte MINA-Brasil, crianças expostas à malária gestacional e amamentadas por pelo menos 12 meses tiveram um risco reduzido de infecção por malária durante os dois primeiros anos de vida^26^. O aleitamento materno protege as crianças pequenas de infecções por transferência passiva de imunoglobulinas e outras substâncias bioativas^26^, o que poderia ser uma possível explicação para a associação de aleitamento materno prolongado e diminuição do risco de anemia aos cinco anos de idade, observada neste estudo.

Até onde sabemos, este é o primeiro estudo a relatar a associação significante entre o consumo de AUP e risco para anemia no primeiro ano de vida. Uma revisão sistemática recente descreveu maior participação da AUP na dieta de crianças associada a outros desfechos materno-infantis, como aumento do ganho de peso, medidas de adiposidade, sobrepeso, desmame precoce, menor qualidade da dieta, alterações metabólicas, doenças e ingestão de plástico das embalagens^27^. Conforme definido pela classificação NOVA de alimentos, AUP são formulações industriais de substâncias derivadas de alimentos com pouco ou nenhum alimento integral com adição de corantes, aromatizantes, emulsificantes, espessantes e outros aditivos cosméticos para torná-los palatáveis ou mesmo hiperpalatáveis^28^. Em um estudo anterior, a introdução do AUP na alimentação complementar associou-se inversamente com a duração do aleitamento materno continuado^29^, fornecendo evidências adicionais para evitar o consumo de AUP na infância.

Este estudo apresenta algumas limitações. Primeiro, os participantes da coorte MINA-Brasil não foram rastreados para infecções helmínticas, distúrbios hemolíticos hereditários e outras condições não nutricionais que podem estar associadas à anemia em ambientes tropicais. Assim, 4% a 10% das crianças amazônicas menores de cinco anos testadas em inquéritos transversais de base populacional apresentam um ou mais helmintos intestinais transmitidos pelo solo que podem causar ou agravar a anemia^22^. No entanto, sua contribuição para a anemia em crianças amazônicas, embora não desprezível, parece ser muito menos pronunciada do que em populações africanas ou asiáticas^22^. A deficiência de glicose-6-fosfato desidrogenase (G6PD), uma doença recessiva ligada ao cromossomo X, é a causa hereditária mais comum de anemia hemolítica na Amazônia. Ela acomete 8,3% da população masculina do Estado do Acre^30^, que pode desenvolver hemólise leve a grave quando exposta a deficiências dietéticas e a alguns medicamentos, incluindo o antimalárico primaquina usado no local. No entanto, a deficiência de G6PD não emergiu como um preditor significativo dos níveis de Hb ou risco de anemia em crianças amazônicas^22^. Em segundo lugar, a prevalência de deficiência de ferro e anemia pode ter sido subestimada nos participantes da coorte MINA-Brasil ao longo do tempo, porque as crianças perdidas no seguimento eram mais propensas a viver nos domicílios mais pobres, estando mais expostas à insegurança alimentar, malária e outros contribuintes para deficiências nutricionais e anemia. Uma série de outras condições (por exemplo, infecções tropicais, inflamação crônica e distúrbios hemolíticos hereditários), algumas delas não abordadas neste estudo, podem ter contribuído para a alta prevalência de anemia em populações amazônicas.

## CONCLUSÃO

Embora nossa população de estudo tenha acesso a cuidados pré-natais, diagnóstico e tratamento imediatos da malária e suplementação de ferro e vitamina A, a anemia em participantes da coorte MINA-Brasil permanece comum durante a gravidez e na primeira infância, especialmente na idade de um ano. Além disso, a DF e o consumo de AUP no primeiro ano de vida foram associados a risco elevado de anemia. Como um problema de saúde pública multifatorial, diferentes possíveis causas devem ser consideradas em todos os esforços e programas para alcançar as metas dos ODS nessa região.
